# Targeting muscle fibrosis using non-specific collagenase injections destabilizes the basal lamina and induces matrix remodeling

**DOI:** 10.1186/s13395-026-00432-7

**Published:** 2026-06-19

**Authors:** Ross P. Wohlgemuth, Sathvik Sriram, Sarah E. Brashear, Kyle E. Henricson, Jason J. Howard, Lucas R. Smith

**Affiliations:** 1 Department of Neurobiology, Physiology, and Behavior, University of California Davis, One Shields Ave, Davis, California 95616 USA; 2 Department of Athletic Training and Clinical Nutrition, University of Kentucky, Lexington, USA; 3 Division of Orthopedic Surgery, Department of Surgery, Izaak Walton Kiliam Health Centre, Dalhousie University, Halifax, Canada; 4 Department of Physical Medicine and Rehabilitation, University of California Davis, Davis, USA

**Keywords:** Skeletal muscle, Muscular dystrophy, Extracellular matrix, Collagenase, Fibrosis

## Abstract

**Background:**

Muscle passive and active function is dependent on the extracellular matrix (ECM). In diseases characterized by muscle fibrosis, namely Duchenne Muscular Dystrophy (DMD), the ECM contributes to deficits in muscle mechanical function and regeneration. Because fibrosis is often viewed as irreversible in DMD and other muscle diseases, there is great incentive to develop anti-fibrotic therapies to prevent or reverse fibrosis.

**Methods:**

In this study we tested the effectiveness of intramuscular injections of non-specific *Clostridium histolyticum collagenase* (CCH) on reducing ECM contents and rescuing muscle mechanical function in D2.*mdx* mice, models of DMD. We performed unilateral injections of collagenase into the tibialis anterior and gastrocnemius in WT and D2.*mdx* mice. We measured in vivo plantarflexion strength, ex vivo muscle mechanical function, immunohistochemistry, and total and cross-linked collagen content.

**Results:**

We found that crude CCH was effective at digesting the muscle ECM but did not provide a therapeutic benefit evidenced by induction of muscle weakness and bleeding within 24 h after CCH injections, and a thickened basal lamina after 7 days.

**Conclusions:**

We conclude that future studies testing collagenase as an anti-fibrotic should use a collagenase specific to fibrillar collagens and a paired physical therapy protocol to better preserve muscle function while reducing fibrosis.

**Supplementary Information:**

The online version contains supplementary material available at https://doi.org/10.1186/s13395-026-00432-7.

## Background

In healthy skeletal muscle, the extracellular matrix (ECM) is vital to active and passive mechanical function [1–3] as well as tissue regeneration [4, 5]. Collagen fibers, the primary load bearers of the muscle ECM [6–9], are integral to muscle mechanical function. Within the muscle ECM, fibrillar collagens provide passive tension [10], transduce contractile force [11], and provide a scaffold for regeneration [5]. However, in cases of disease such as Duchenne Muscular Dystrophy (DMD) and Cerebral palsy (CP), the muscle ECM undergoes changes in content and architecture that lead to ECM accumulation or fibrosis. Muscle fibrosis can negatively affect muscle mechanical function [12, 13] and satellite cell function [4], which together make tissue regeneration progressively more difficult. In neuromuscular disorders such as DMD, highly cross-linked collagen fibers replace a portion of the volume allocated to muscle fibers, increasing passive muscle stress and decreasing contractile force [14–16]. Additionally, this increased fibrotic volume is difficult to turn over, accumulating over time [6, 17, 18]. As a result, there is a clear need for anti-fibrotic treatments that address the increased intramuscular connective tissue inherent to a majority of neuromuscular diagnoses. A potential anti-fibrosis treatment is Collagenase *Clostridium histolyticum* (CCH), which is an FDA-approved compound used in the treatment of Dupuytren’s contracture [19, 20] and Peyronie’s disease [21, 22]. Used in these conditions, CCH targets dense, collagen-rich cords to reduce their stiffness [23, 24] and improve tissue compliance [19]. Its favorable balance of safety and clinical benefit in these fibrotic conditions supported its regulatory approval in the United States, where it is marketed as Xiaflex (Endo Pharmaceuticals, Malvern, PA). There is also a growing body of evidence showing the capability of reducing ex vivo muscle stiffness using CCH [6, 25–27]. In DMD and CP, the development of muscle contractures is related to increased muscle stiffness [28, 29]. If CCH could be effective in digesting fibrotic ECM within muscle, it could slow the development of muscle contractures and provide a large benefit to people suffering from muscle fibrosis [27, 30]. Thus, we sought to test the effectiveness of CCH in reducing fibrosis and stiffness within dystrophic muscles. The aim of our study was to assess the efficacy of CCH at reducing muscle fibrosis and stiffness in the D2.*mdx* mouse, a model of DMD. Although the muscle ECM is primarily of fibrillar collagens, namely type I and III collagen [3, 7], we selected a non-specific CCH that targets fibrillar and non-fibrillar collagens to exert a large effect on the ECM, rather than preserve its function. Our results demonstrate that intramuscular injection of non-specific CCH digests collagen IV networks within the basal lamina, impairing contractile function and inducing intramuscular bleeding. These findings highlight the negative impact of targeting non-fibrillar collagens with CCH but provide evidence that administering CCH specific to fibrillar collagens could be a potential anti-fibrotic therapy.

## Methods

### Sex as a biological variable

We used mice of both sexes in this study (wildtype male *N* = 7, wildtype female *N* = 4, D2.*mdx* male *N* = 9; D2.*mdx* female *N* = 9). We did not expect sex-related differences in our primary outcomes and did not observe significant sex-related differences in our data. Our findings are applicable to both sexes.

### Animal handling and footplate torque

DBA/2J (wildtype) and D2.B10-Dmd*mdx*/J (D2.*mdx*) mice were cared for in the UC Davis Teaching and Research Animal Care Services Facility. Mice were housed on a 12:12 light-dark cycle and provided *ad libitum* access to food and water. Mice were between the ages of 34–38 weeks at the time of injections and sacrifice. Prior to intramuscular injections mice were placed under anesthesia with 2.5% Isoflurane gas in 1 L/min oxygen. While under anesthesia, plantarflexion torque at the ankle was measured in both hindlimbs using a 305 C-LR Dual Mode Muscle Lever and Footplate system (Aurora Scientific). Hindlimbs of anesthetized mice were treated with depilatory cream to remove hair prior to electrode placement. After hair removal, the right foot was attached to a footplate at approximately 90° knee flexion and 90° ankle flexion. The right foot was then adhered to the footplate using masking tape to prevent slippage during in vivo mechanical testing. The skin around the gastrocnemius and electrodes were disinfected with 70% ethanol wipes. Electrodes were then inserted into the mid-belly of the gastrocnemius. Single muscle twitches were induced using 40mV electrical stimulations and electrodes were reoriented to maximize plantarflexion torque and minimize co-contraction of antagonistic muscle groups. Once electrodes were properly oriented, a force frequency curve from 20 to 150 Hz was generated with 10 s of rest in between tetani. Torque was reported as the amplitude of the largest tetanus normalized to bodyweight and the moment arm of the footplate. The left limb was immediately tested following measurement of right ankle torque. Footplate torque was measured on the day of the intramuscular injection and the day of sacrifice, which took place 1-, 3-, or 7-days post-injections.

### Intramuscular injections

**Fig. 1:**
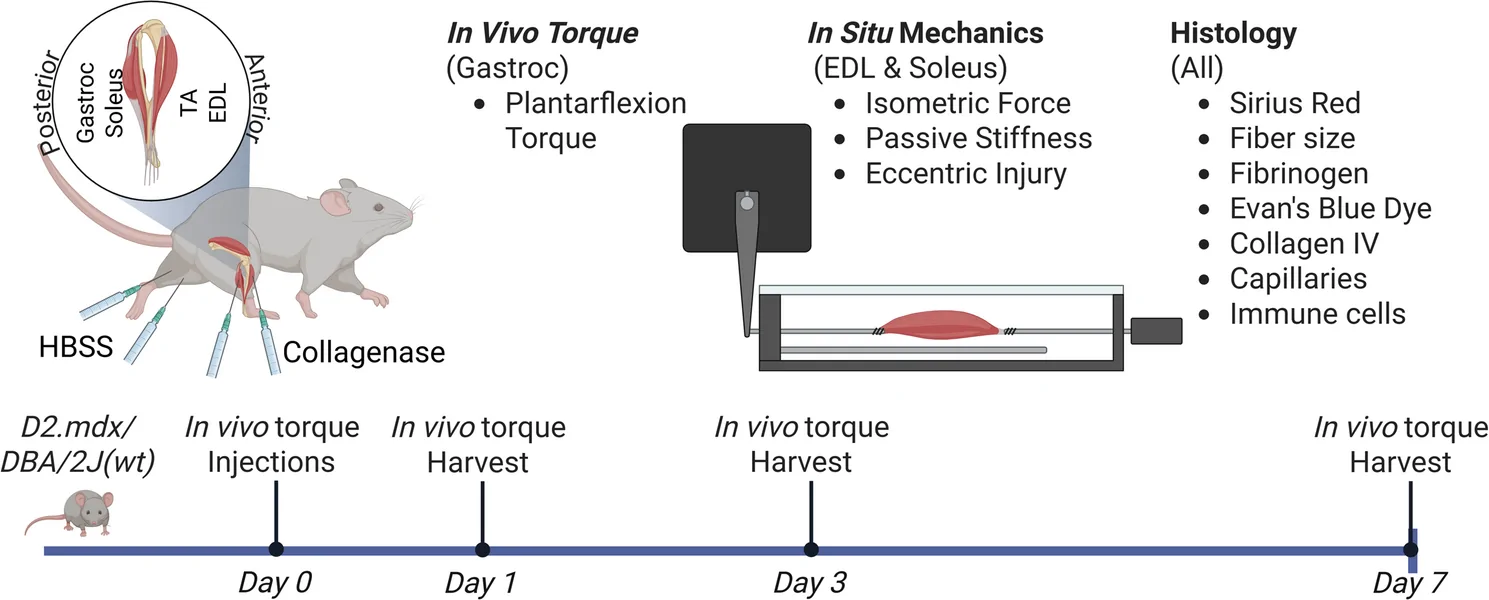
Overview of experimental design. Wildtype (DBA/2J) and dystrophic (D2.*mdx*) mice were used in experiments. Each hindlimb received HBSS (Hank’s Balanced Salt Solution, saline) or collagenase *Clostridium histolyticum* (CCH) intramuscular injections into gastrocnemius (GA) and tibialis anterior (TA) muscles. Mice were sacrificed and tissues were collected 1-, 3-, or 7-days following CCH injections. Primary outcome measures are listed

Following the initial measurement of ankle plantarflexion torque on both hindlimbs, each mouse was kept under anesthesia and received two intramuscular injections on each hindlimb using a 28 gauge 0.5 ml syringe. Each hindlimb received a separate intramuscular injection into the muscle belly of the gastrocnemius and the tibialis anterior muscle, respectively, for a total of four intramuscular injections per mouse (Fig. 1). Each mouse received two injections of saline (Hank’s Balanced Salt Solution with calcium and magnesium; Gibco, Carlsbad, CA) in one hindlimb and two injections of collagenase (0.1 mg of type II collagenase *Clostridium* histolyticum; Gibco, Carlsbad, CA) dissolved in 50µL of HBSS (Hank’s Balanced Salt Solution, saline) in the contralateral hindlimb. The dose of collagenase was selected based on previous reports [23, 24, 27]. Following intramuscular injections mice were removed from anesthesia and returned to their cage. Mice were monitored for 30 min until they were alert and ambulatory. Food and water were provided on the ground of the cage for up to three days following intramuscular injections. Analgesics were administered if either injected leg exhibited lameness for more than 24 h post-injections.

### Animal dissection and muscle isolation

On the day of collection, mice were anesthetized with 2.5% Isoflurane gas in 1 L/min for muscle isolation. Muscles from the hindlimbs including the tibialis anterior (TA), extensor digitorum longus (EDL), gastrocnemius (GA), soleus, and quadriceps (QU) were collected while the mice were under anesthesia. Following collection of muscles, cervical dislocation was performed while mice were under anesthesia. Heart, liver, and tibias were collected after sacrifice and flash frozen in liquid nitrogen. Soleus, EDL, and TA muscles were stored in a dish of oxygenated Ringer’s solution (Sodium Chloride, Potassium Chloride, Calcium Chloride Dihydrate, Potassium Phosphate Monobasic, Magnesium Sulfate, 4-(2-Hydroxyethyl)piperazine-1-ethanesulfonic acid (HEPES), Glucose). GA and QU muscles were pinned on cork and embedded in Optimal Cutting Temperature (OCT) solution before flash freezing in liquid nitrogen cooled isopentane. Flash frozen muscles were stored at -70 °C.

### Ex vivo muscle passive mechanical testing

Soleus and EDL muscles were prepared for ex vivo passive mechanical testing as previously described [6, 13, 14, 26]. In short, 7 − 0 sutures were tied at the myotendinous junctions of the soleus and EDL muscles. Loops of suture were placed on hooks attached to the 300 C-LR-Dual-Mode motor arm and force transducer (Aurora Scientific) such that the muscle sat within 28 °C oxygenated Ringer’s solution. Twitches were induced using a 701 C stimulator (Aurora Scientific) across a range of muscle lengths to determine the optimal length for isometric force generation (L_o_). The L_o_ length corresponded to the distance between the sutures on either myotendinous junction, measured by calipers. Physiological cross-sectional area (PCSA) was calculated using the muscle length (L_m_), mass (m), ratio of fiber length to L_o_ (L_f_/ L_o_) and standard density of muscle (ρ = 1.06 g/cm^3^; PCSA = m/ L_o_ *(L_f_/ L_o_)*ρ) [31].

Soleus and EDL muscles underwent passive mechanical testing as previously described [6, 13, 14, 26]. In short, muscles started at L_o_ and were subjected to a series of stretch-shortening cycles and held stretch steps. Stretch-shortening cycles occurred before each held stretch step and entailed stretching and shortening the muscle by 2.5% relative to L_o_ at a rate of 1 Hz for 5 s. Held stretch steps entailed stretching the muscle by 2.5% beyond L_o_ at a rate of 1 L_o_/s and maintaining the muscle at the new length for 120 s. Stretch-shortening cycles and held stretch steps were repeated up to 5, 7.5, 10, and 12.5% strains using increments of 2.5% of L_o_. The passive stiffness was reported as the slope of the quadratic fit to the plot of stress (tensile force per PCSA) and strain data. Dynamic stiffness was the slope of the fit with the maximum stress values over the held stretch steps while elastic stiffness was the slope of this fit with stress values recorded at the end of the held stretch steps. Elastic index was reported as the ratio between elastic and dynamic stiffness.

TA muscles underwent a different passive mechanical testing protocol due to the difficult in inducing muscle twitches without an accessible proximal tendon following dissection. Since L_o_ was not measured for the TA, the starting length (L_s_) was set as the minimum length at which the TA muscle was completely straightened on the mechanical testing apparatus. This was set by placing the tied TA muscle in the Ringer’s bath and applying a strain to let the TA straighten out in line with the 300 C-LR-Dual-Mode motor arm. Once the TA was straightened out and began to visibly stretch with increasing strain, the length between the sutures on either end of the TA was measured and recorded as the starting length. TA muscles were then subjected to stretch-shortening cycles and held stretch steps like the EDL and soleus, except across a range of strains from 5%, 10%, 15%, 20%, and 25% of Ls in 5% increments. PCSA was calculated like the EDL and soleus except L_o_ was replaced with L_s_. TA muscles were pinned on cork, embedded in OCT, flash frozen in liquid nitrogen cooled isopentane, and stored at -70 °C.

### Ex vivo active mechanical testing

Soleus and EDL muscles were subjected to active mechanical testing following passive mechanical testing. In between passive and active protocols, the bath of 28 °C oxygenated Ringer’s solution was replaced with a mixture of 0.2% Evans Blue Dye (EBD) (Millipore Sigma, Burlington, MA) dissolved in 28 °C oxygenated Ringer’s solution. EBD was used to detect muscle fibers that encountered damage to the sarcolemma during the active mechanical testing. Active mechanical testing began with a maximum isometric twitch (300 mA, 1.2 ms pulse width), pausing for 30 s of rest, and measuring maximum isometric tetanus (300 mA, EDL: 0.3 ms pulse width, 120 Hz pulse frequency, 300 ms pulse train; soleus: 0.3 ms pulse width, 80 Hz pulse frequency, 800 ms pulse train). Muscles were then given 120 s of rest before a series of five eccentric contractions. Eccentric contraction bouts consisted of a series of two isometric contractions divided by an active stretch-shortening cycle to 1.1 L_o_ (10 L_o_ /s, 20 ms) at 500 ms after the start of electrical stimulation (300 mA, EDL: 0.3 ms pulse width, 120 Hz pulse frequency, 800 ms pulse train; soleus: 0.3 ms pulse width, 80 Hz pulse frequency, 800 ms pulse train). Muscles rested for 120 s in between eccentric bouts. Following the fifth eccentric bout, 120 s were given for the muscle to rest before repeating the same maximum isometric twitch and tetanus protocol that started the active mechanical test. Maximum isometric force was measured during the first and last tetanus protocol and during the initial isometric tetani that took place prior to the active stretch-shortening cycle in each eccentric bout. Maximum eccentric force was taken as the highest force reading during the eccentric bout, which occurred during the active stretch-shortening cycle. Maximum forces were normalized to PCSA to give isometric and eccentric specific tension. The highest isometric specific tension measured during each active protocol was reported as the isometric specific tension for each muscle. After active mechanical testing was completed, muscles were removed from the mechanical testing equipment and the Achilles tendon was cut away from the soleus. EDL and soleus muscles were pinned on cork, embedded in OCT, flash frozen in liquid nitrogen cooled isopentane, and stored at -70 °C. Achilles tendons were flash frozen in liquid nitrogen and stored at -70 °C.

### Immunohistochemistry and fluorescent imaging

Soleus, EDL, TA, and GA muscles were sectioned at -20 °C into 10 μm transverse slices. Sections were adhered to glass slides and stored at -20 °C until staining was performed. Muscle sections were circled with a hydrophobic PAP pen, fixed with 4% paraformaldehyde (PFA) for 15 min, and washed three times for 5 min with phosphate buffered saline (PBS). 0.5% Triton was used on the sections for 10 min to lyse the cells. The sections were then blocked with 0.1% bovine serum albumin (BSA) two times for 5 min. Then 5% BSA was applied to the sections for 30 min prior to primary antibody application. Primary antibodies were diluted in 5% BSA and applied to the sections for an overnight incubation at 4 °C. Sections that received a primary antibody that contained a fluorescent conjugate were kept in the dark for the primary incubation and the remainder of the protocol. Primary antibody antigen, host, and dilution are listed in Table 1. Following overnight incubation, sections were washed three times for 5 min in 0.1% BSA. Secondary antibodies diluted in 0.1% BSA were added to sections for a 90-minute incubation in the dark. Slides were maintained in the dark for the rest of the protocol. Secondary antibody antigen, host, and dilution are listed in Table 1. Following secondary incubation, sections were washed three times for 5 min in 0.1% BSA. For sections that required visualization of nuclei, a Hoechst stain was applied (1:2000; Fisher H3570) for 15 min. Sections were then washed twice for 5 min in 0.1% BSA and then once more in PBS for 5 min. Prolong Gold (Fisher P36934) was added to the sections and a glass coverslip was adhered to the top of each slide.

**Table 1:** Antibody information

Antibody name	Manufacturer	Product number	Dilution
WGA-488*	Fisher	W6748	1:200
Anti-Fibrinogen	Agilent	A008002-2	1:200
Anti-CD45	Biolegend	103,102	1:100
Anti-CD31/PECAM-1	Santa Cruz Biotechnology	Sc-376,764	1:50
Anti-Collagen IV	Abcam	Ab6586	1:100
Anti-rat 660	ThermoFisher	50-4815-82	1:100
Anti-rabbit 555	Fisher	A32794	1:500

*Wheat germ agglutinin (WGA) is not an antibody-based reporter

Immunostained sections were imaged by epifluorescence using an inverted Leica DMi8 microscope. Each section was imaged by a tile scan using a 20X objective. DAPI, FITC, Rhodamine, and Cy5 channels were used to image muscle sections. Tile scans were merged for image analysis. Images were analyzed for signal thickness, percent positive area, number of capillaries per fiber, fiber size, central nuclei, and EBD-positive fibers. Each set of slides was imaged using identical settings including intensity and exposure. Custom MatLab scripts and ImageJ macros were used for histological quantification.

### Sirius red staining and imaging

Picrosirius red staining and imaging was performed as previously described [6, 13, 32–34]. Soleus, EDL, TA, and GA muscle sections were circled with a hydrophobic PAP pen and fixed in 4% PFA for 10 min. Sections were then washed with deionized water three times for 5 min and left out to dry for 15 min. Sections were stained with picrosirius red solution for 60 min and washed two times in acidified water for 1 min each. Sections were dehydrated using three washes of 100% ethanol for 1 min each. Citrisolv was added for 3 min and Permount was applied to the sections before a glass coverslip was adhered to the slide.

Transverse muscle sections were imaged using brightfield illumination and a 20X objective on a Leica DMi8 microscope and DFC9000GTC camera. Linearly polarized light imaging was used with a rotating polarizer in the light path before and after the sample. Sirius red area and collagen fiber density were quantified using a custom MatLab script (Mathworks, Natick, MA) as previously described [6]. For collagen density quantification, pixels were sorted by color to measure the density of associated collagen fibers. Green, yellow, and red colored pixels were considered to represent loose, intermediate, and high collagen packing density, respectively.

### Hydroxyproline and collagen solubility assay

GA, TA, and QU were analyzed for total and insoluble (cross-linked) collagen content using the hydroxyproline and collagen solubility assay as previously described [6, 13, 14, 26, 34, 35]. GA and TA muscles embedded in OCT were removed from − 70 °C storage, melted out of OCT, washed in PBS, and flash frozen in liquid nitrogen. All samples were powdered on dry ice with mortar and pestle. Powdered tissue was weighed and mixed in PBS at 4 °C for 30 min. Samples were then spun at 21,000 g for 30 min at 4 °C. The supernatant was removed, and pellets were placed in 0.5 M Acetic Acid with 1 mg/ml pepsin and incubated with agitation overnight at 4 °C. Samples were then spun for 30 min at 21,000 g at 4 °C before separation of the pellet (insoluble fraction) and supernatant (soluble fraction). The soluble fraction was boiled on a hot plate at 110 °C and both fractions received 0.5 ml of 6 N Hydrochloric acid. A set of hydroxyproline standard solutions (0–1,000 µM trans-4-hydroxy-_L_-proline; Fisher) also received 0.5 ml of 6 N Hydrochloric acid. Solutions were mixed and boiled overnight at 110 °C to induce hydrolysis of collagen peptides. Following hydrolysis, 10µL of each sample and standard was aliquoted into a new tube for the rest of the protocol. 150µL of isopropanol was added to each tube along with 75 µL of solution A (1:4 dilution of 7% chloramine T (ThermoFisher) in water to acetate citrate buffer) before mixing and leaving at room temperature for 10 min. 1 ml of solution B (3:13 dilution of Erlich’s reagent to isopropanol) was added to each tube before mixing and placing in a 58 °C hot plate for 30 min. Tubes were then removed from heat and placed on ice before centrifugation at 5,000 g at 4 °C for 1 min. 100µL from each tube was transferred to a 96-well plate in duplicate for measurement of absorbance at 558 nm. Total and insoluble collagen content per tissue mass was calculated using the hydroxyproline standard absorbance curve and reported as µg collagen per mg tissue.

### Statistical methods

Statistics were performed in GraphPad Prism (version 10.3.1). Mixed effect analyses were run in lieu of analyses of variance (ANOVA) when data points were missing due to mechanical failure during passive or eccentric protocols. Three-way ANOVAs or mixed effect analyses were run on specific tension across the eccentric protocols by tetanus number, genotype, and collagenase. Two-way ANOVAs or mixed effect analyses were run on mechanical, histological, and biochemical data across each date of collection by genotype and collagenase. Two-way ANOVAs were run on body weight by genotype and time. Post-hoc Sidak, Tukey, or Fisher’s least squared differences tests were run based on significant effects determined by mixed effect analysis or ANOVA. All data are reported as mean ± SD unless otherwise stated.

### Study approval

All experiments involving animals were approved by the University of California Davis Institutional Animal Care and Use Committee under IACUC protocol #22,579.

## Results

### CCH injections did not decrease passive stiffness

**Fig. 2:**
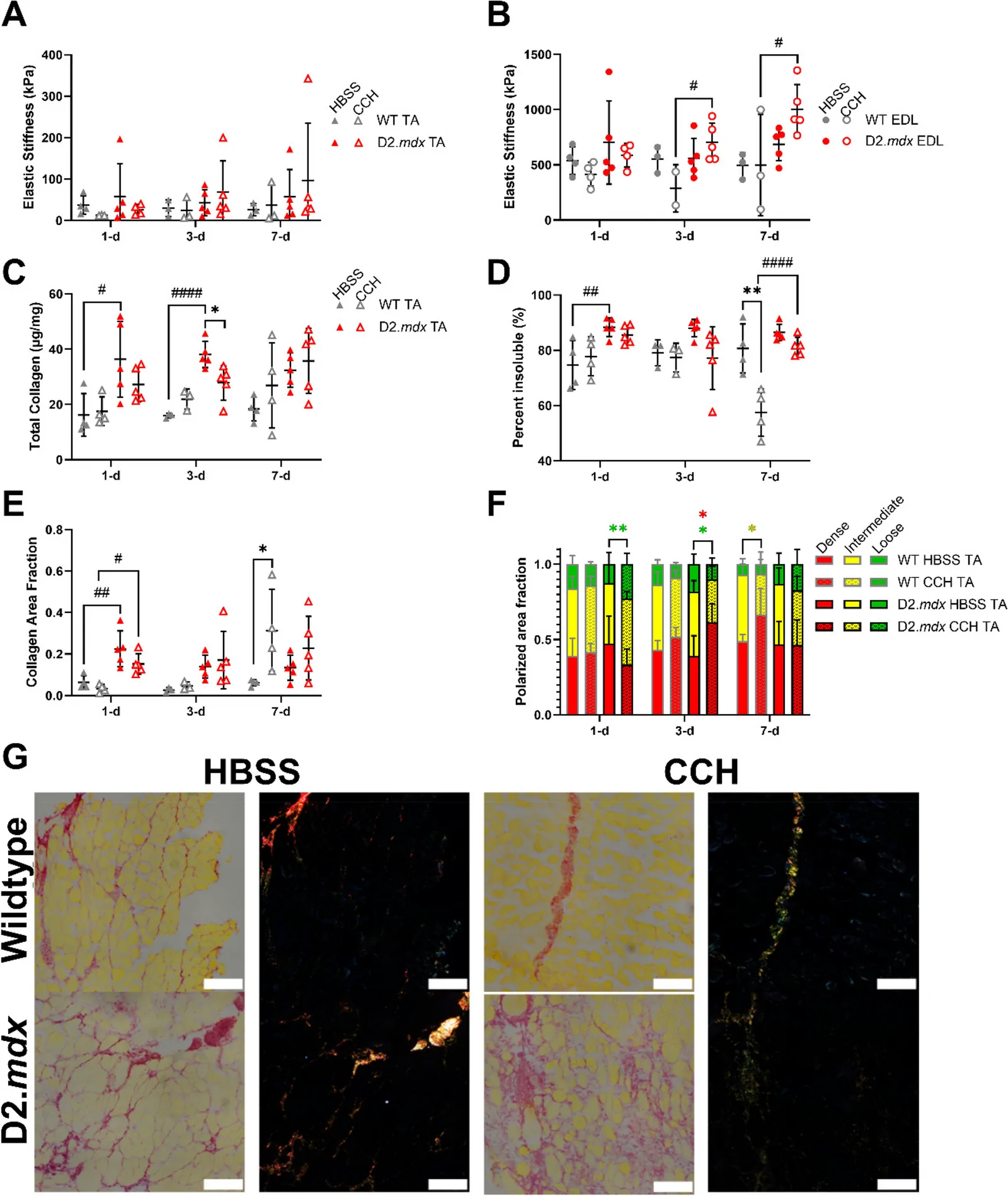
Passive muscle mechanics and collagen matrix characteristics following CCH. **A** Elastic stiffness, measured at 20% strain from slack length, was not significantly different between groups for the tibialis anterior (TA) muscle. **B** Elastic stiffness, measured at 10% strain from Lo, was higher in CCH injected extensor digitorum longus (EDL) muscles from D2.mdx mice compared to WT at 3- and 7-days post-injection. **C** Total collagen content was increased in D2.mdx TAs compared to WT at 1- and 3-days after CCH injection. CCH injection reduced collagen content in D2.mdx TAs compared to saline injected after 3 days. **D** D2.mdx TAs had a greater degree of cross-linking than WT at 1- and 7-day(s) after CCH. CCH injection reduced the degree of collagen cross-linking after 7 days. **E** D2.mdx TAs had higher collagen area fractions than WT at 1-day post-injection. CCH injected TAs had a higher collagen area fraction than saline after 7 days. **F** CCH injections reduced collagen packing density in D2.mdx TAs after 1 day, but increased density after 3 days. CCH injected WT TAs had less intermediately packed collagen than saline injected after 7 days. **G** Representative Sirius Red stained TA cross-sections under brightfield and polarized light at 1-day post-injections. Scale bar=200 μm. Bracketed bars represent significance by Sidak’s multiple comparisons post-hoc tests. Significance by CCH: *p < 0.05, **p < 0.01; significance by genotype: #p < 0.05, ##p < 0.01, ####p < 0.0001

Following CCH intramuscular injections, we did not observe a significant decrease in passive stiffness in any muscle tested (Fig. 2A-B; Supplemental Fig. 1A). CCH injected D2.*mdx* EDL muscles had greater stiffness than the CCH injected WT muscles on days 3 and 7 (Fig. 2B). In parallel, CCH injected soleus muscles were stiffer than saline (Hank’s Balanced Salt Solution, HBSS) injected 7-days post-injection (Supplemental Fig. 1A). Thus, CCH injections were not sufficient to appreciably reduce passive muscle stiffness.

### CCH injections acutely reduced collagen content

Using the hydroxyproline and collagen solubility assays and picrosirius red staining, we analyzed the changes to fibrillar collagen within muscle in response to CCH injection. We observed multiple significant effects of CCH on collagen content, cross-linking, and density in the TA muscles. We found that CCH reduced collagen content in D2.*mdx* TA muscles 3 days following injection, but this difference was absent by 7 days (Fig. 2C). CCH was also effective at reducing cross-linked collagen in the WT TA at 7 days post-injection (Fig. 2D). D2.*mdx* TA muscles had more collagen content, cross-links, and collagen area than WT at multiple time points (Fig. 2C-E). In D2.*mdx* TA muscles, CCH injection led to an early increase in loosely packed collagen that was absent within 3 days (Fig. 2F-G). In the gastrocnemius (GA), CCH injections did not alter collagen content, cross-linking, or area fraction (Supplemental Fig. 2A-B). However, we did observe increases in collagen cross-linking and area fraction in D2.*mdx* GA muscles compared to WT, as expected (Supplemental Fig. 2B, E-G). To assess the localization of the effects of CCH, we measured collagen content in the quadriceps muscles, which did not receive CCH. We found largely no CCH induced changes in collagen content and cross-linking in the quadriceps (Supplemental Fig. 2C-D), consistent with localized effect of CCH. Based on these findings, it is evident that CCH was not effective at reducing fibrosis in the long term.

### CCH injections were detrimental to contractile function independent of fiber size

**Fig. 3:**
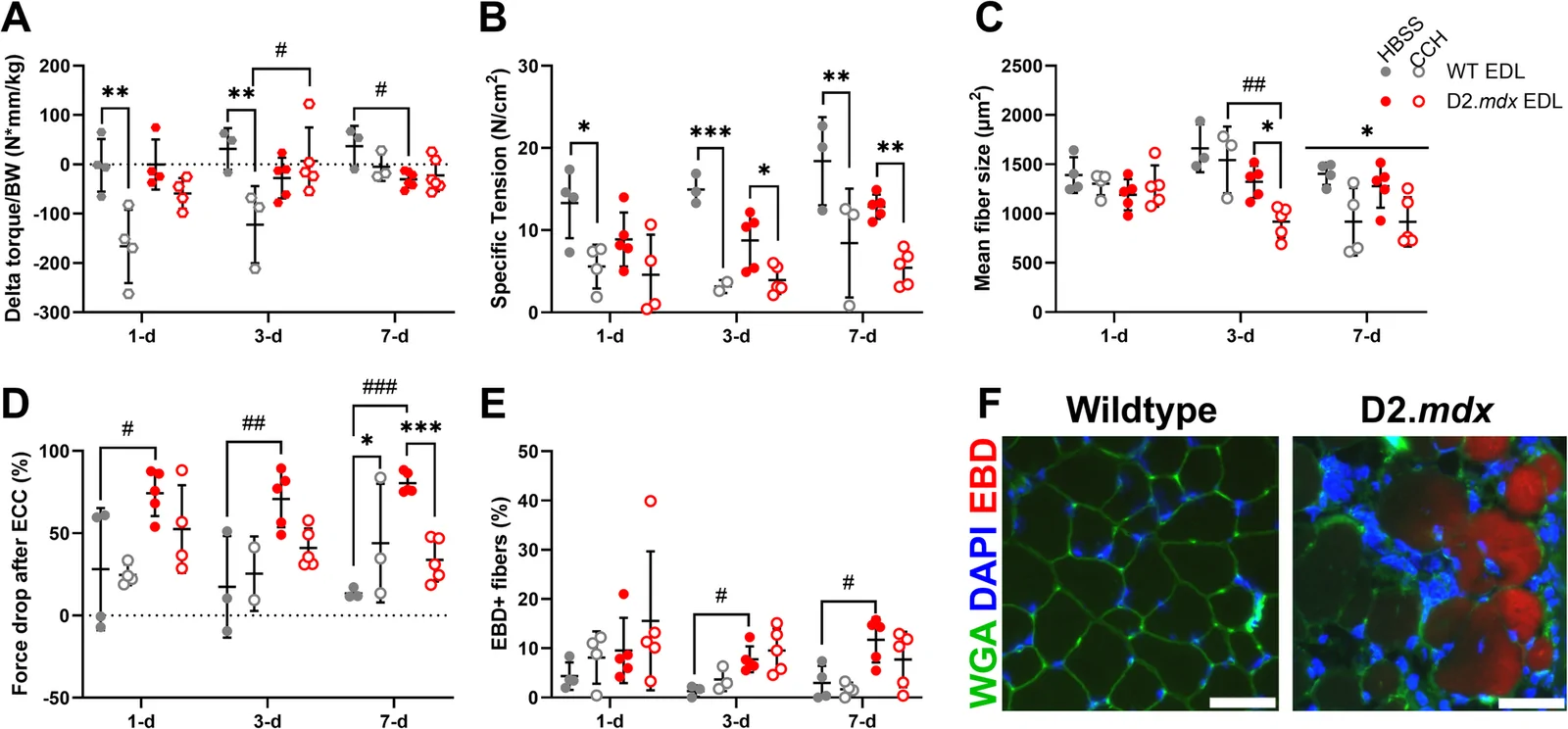
Muscle strength, fiber size, and damage susceptibility following CCH. **A** The drop in GA normalized torque from 0- to 1- and 3-days post-injection were more negative in the WT CCH injected limb than the saline injected limb. The drop in torque in CCH injected limbs tended to be larger in WT mice than in D2.mdx. **B** CCH injections consistently reduced specific tension of EDL muscles. **C** CCH injections reduced myofiber size in EDLs after 3 days. **D** D2.mdx EDLs lost more force by the end of the ECC protocol than WT. **E** D2.mdx EDLs had a greater proportion of Evans Blue Dye (EBD) positive myofibers than WT EDLs at 3- and 7-days post-injection. **F** Representative images of EDL muscles one day following ECC protocols. Scale bar=50 μm. Flat bars represent significant main effects from two-way ANOVAs or mixed effect analyses. Bracketed bars represent significance from Sidak’s multiple comparisons post-hoc tests. Significance by CCH: *p < 0.05, **p < 0.01, ***p < 0.001; significance by genotype: #p < 0.05, ##p < 0.01, ###p < 0.001, ####p < 0.0001

Throughout the time course of our experiments, we did not observe any concerning decreases (> 20%) in body mass (Supplemental Fig. 1C). We found that the WT GA had greater normalized muscle mass after 1- and 3-days after CCH injections, while TA muscles did not exhibit significant differences in mass (Supplemental Figs. 1E-F). To assess whether CCH decreased muscular strength in vivo, we measured the maximum isometric torque produced by the ankle plantar flexors. We found that saline injections did not change plantarflexion torque across the time course (Fig. 3A; Supplemental Fig. 1D). However, CCH induced early declines in torque in WT mice that returned to baseline by 7-days post-injection (Fig. 3A; Supplemental Fig. 1D). Across all time-points the D2.*mdx* mice produced less torque than the WT mice (Supplemental Fig. 1D). After sacrifice, we performed active muscle mechanics on EDL and soleus muscles ex vivo. We found that CCH consistently reduced the specific tension of EDL muscles across the time course (Fig. 3B). This observation was similar in the WT soleus muscle (Supplemental Fig. 1B). Next, we assessed the histological changes in muscle fiber size and regeneration in response to CCH. We found that CCH reduced muscle fiber size within 3–7 days of injection (Fig. 3C; Supplemental Fig. 3A-K). We also measured centrally nucleated fibers to assess the regeneration state of muscle through the time course post-collagenase injection. We found that D2.*mdx* muscles tended to have higher proportions of central nucleated fibers than WT, as expected [36, 37] (Supplemental Fig. 4A-C). Interestingly, we found that CCH decreased the prevalence of centrally nucleated fibers at various time points in the TA, EDL, and soleus (Supplemental Fig. 4A-C). These findings indicate that CCH reduces muscle strength prior to a change in fiber size.

### CCH injections did not increase eccentric contraction induced damage

To quantify the impact of CCH on sarcolemma integrity, we induced a series of lengthening (eccentric) contractions on EDL and soleus muscles ex vivo. We added Evans Blue Dye (EBD) to the bath of Ringer’s solution to measure incorporation of EBD into damaged myofibers. Unsurprisingly, we found that D2.*mdx* EDL muscles were more susceptible to eccentric contraction induced force loss than WT [38] (Fig. 3D, Supplemental Fig. 5A-C). We also found that CCH reduced the eccentric-induced force drop in the EDL, although the baseline force started lower than saline injected controls (Fig. 3D). In parallel, there was a higher proportion of EBD-positive fibers in D2.*mdx* EDL muscles compared to WT at days 3 and 7, but no significant effect of CCH (Fig. 3E-F). In the soleus, we found less effects of dystrophy, as expected [39], and CCH (Supplemental Fig. 5D-G). Overall, contrary to our hypothesis we found that CCH weakened skeletal muscles but did not make them more susceptible to further damage from eccentric contractions.

### CCH injections degraded collagen IV networks and induced intramuscular bleeding

**Fig. 4:**
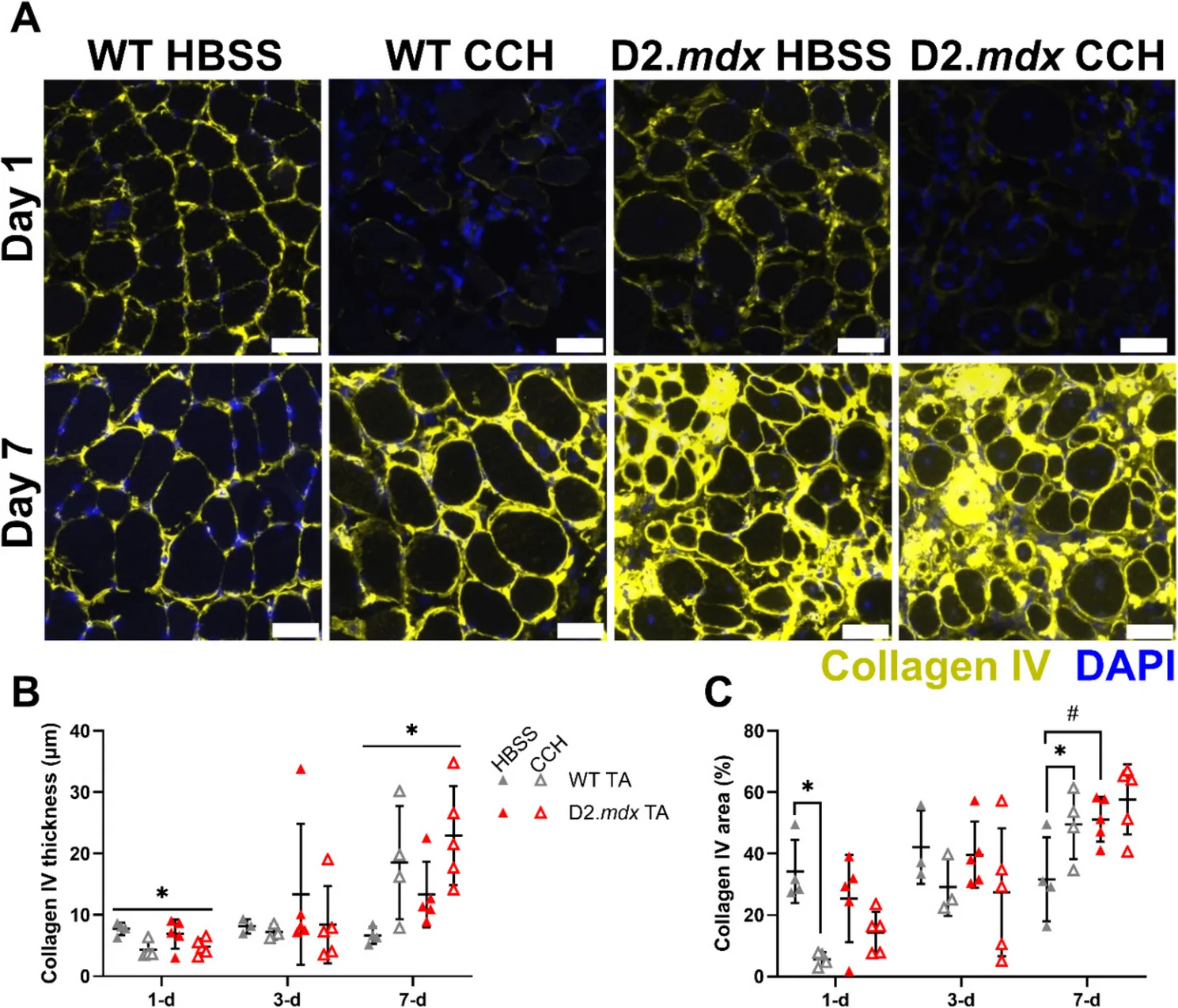
CCH acutely ablates collagen IV networks, which thicken one week after injections. **A** Representative images of collagen IV layers around TA muscle fibers. **B** CCH injected TAs had reduced collagen IV layer thickness after 1 day, but increased layer thickness after 1 week. **C** CCH injected TAs had reduced collagen IV area after 1 day, but increased collagen IV area after 1 week. Scale bar=50 μm. Flat bars represent significant main effects from two-way ANOVAs or mixed effect analyses. Bracketed bars represent significance by Sidak’s multiple comparisons post-hoc test. Significance by CCH: *p < 0.05; significance by genotype: #p < 0.05

To assess effects of CCH on non-fibrillar collagens, we expanded our analysis to collagen IV (Fig. 4A), the major collagen of the basal lamina in muscle and blood vessels [40]. We observed that the layer of collagen IV around muscle fibers was initially thinner with CCH on day 1 but became thicker by day 7 post-injection (Fig. 4B). Similarly, the total area of collagen IV within the TA was reduced by CCH on day 1 but was elevated on day 7 (Fig. 4C). From these data it is evident that CCH degrades the collagen IV network within the basal lamina early on and induces an ECM remodeling response that increases fibrillar collagen area, basal lamina thickness, and soluble collagen content by day 7 post-injections. Thus, CCH effectively degrades muscle ECM early on but leads to basal lamina thickening and a weakened ECM that hinders muscle mechanical function.

**Fig. 5:**
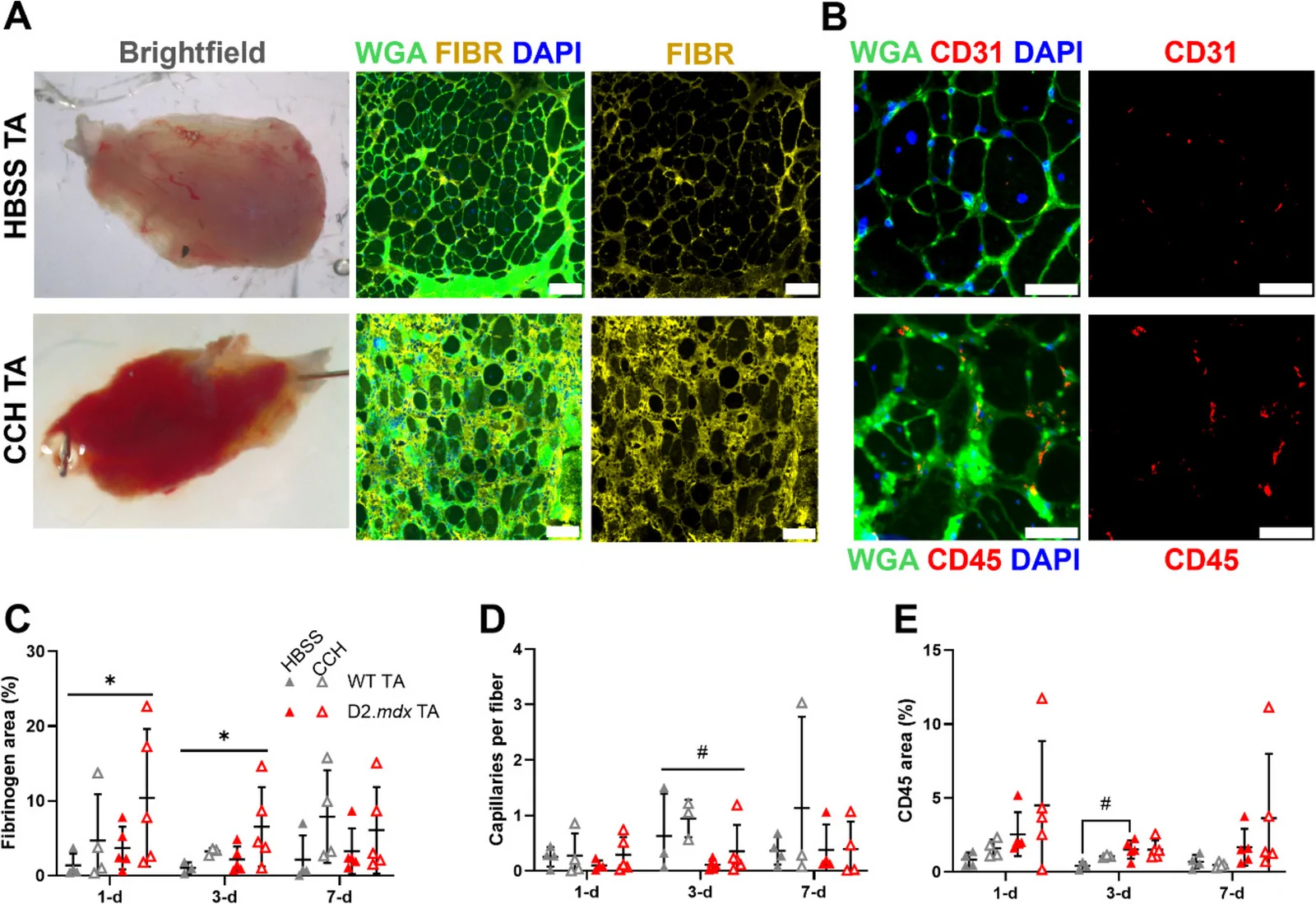
CCH induces intramuscular bleeding independent of inflammatory markers. **A** Representative images of saline and CCH injected TA muscles showing intramuscular blood clots. Fluorescent microscopy shows increased fibrinogen (FIBR) positive area in CCH injected TA muscles compared to HBSS (Hank’s Balanced Salt Solution, saline) injected. Scale bar=100 μm. **B** Representative images for CD45 and CD31 in cross-sections of muscle. Scale bar=50 μm. **C** CCH injected TA muscles had significantly increased fibrinogen positive area at 1- and 3-days after injection. **D** Number of capillaries per muscle fiber was higher in WT TA muscles on day 3 compared to D2.mdx. **E** D2.mdx muscles had greater CD45% area than WT at 3 days following CCH. Flat bars represent significant main effects from two-way ANOVAs or mixed effect analyses. Bracketed bars represent significance from Sidak’s multiple comparisons post-hoc tests. Significance by CCH: *p < 0.05, **p < 0.01; significance by genotype: #p < 0.05

During sacrifice and dissection, we repeatedly noticed that muscles from the CCH injected limb appeared a blood red hue compared to the saline injected limb even after repeated rinsing in Ringer’s solution (Fig. 5A). We anticipated that this color change was due to increased blood within the muscle belly. Thus, we investigated the effects of CCH on the intramuscular vasculature and immune cell infiltration. We observed that CCH increased the fibrinogen-positive area, a marker of intramuscular bleeding [41], within TA and soleus muscles at days 1 and 3, and EDL muscles at days 3 and 7 post-injection (Fig. 5B-C; Supplemental Fig. 4D-E). However, this was not paralleled by CD45 + cell infiltration in CCH injected TA muscles (Figs. 5B, E). We also investigated whether CCH injection altered muscle vascularity using a CD31 antibody for vascular endothelial cells. However, we did not find any significant differences in capillaries per myofiber (Fig. 5B, D). Thus, we concluded that CCH injections increased bleeding within the muscle tissue but did not alter immune cell numbers or blood vessel density within the time course examined.

## Discussion

When muscle is severely or chronically injured, there is a robust increase in transcription and synthesis of collagens, matrix metalloproteinases (MMPs), and other matrix components that help remodel the ECM to facilitate muscle regeneration [42–44]. In our study, we attempted to target the fibrillar collagen matrix using non-specific CCH to initiate ECM remodeling that would reduce fibrosis and passive stiffness in dystrophic muscle. We found that while CCH was effective at digesting the muscle ECM, it did not appreciably reduce passive stiffness and was detrimental to muscle mechanical function. Over the course of 7 days CCH acted locally to the site of injections and reduced muscle strength within 24 h prior to a change in fiber size. Muscle passive stiffness was not decreased by CCH, instead passive stiffness was trending higher 7 days following injections. Within 3 days total collagen content declined in the fibrotic TA and within 24 h collagen IV networks around muscle fibers were thinning. By targeting collagen IV, CCH also induced intramuscular bleeding through degradation of blood vessel walls. However, by 7 days muscle ECM remodeling *increased* collagen content and thickened the basal lamina, reversing the initial effects of CCH. Based on our observations, we found that intramuscular injection of non-specific CCH did not provide a therapeutic benefit to fibrotic muscle, however, this does not preclude the possibility that specific CCH compounds could be effective.

We expected that CCH would be effective at reducing muscle passive stiffness in vivo based on the dramatic effects of CCH on muscle ex vivo [6, 25, 26]. Surprisingly, we did not observe a consistent reduction in passive stiffness related to CCH in any muscle tested. In fact, we observed that EDL and soleus muscles tended to have increased passive stiffness one week after CCH injections. Previous experiments that have exposed muscles to collagenase ex vivo have reported large reductions in passive stiffness following just one hour of collagenase treatment [6, 25, 26]. These experiments also reported little to no change in total collagen content, showing that the digestion of the collagen matrix rather than a change in collagen content, was sufficient to reduce passive stiffness in muscle. In our study, we demonstrated that the D2.*mdx* TA muscle had decreased total collagen content at 3 days following CCH, without an associated decline in passive stiffness. While both fibrillar and non-fibrillar collagens were targeted to some degree, our non-specific CCH may have preferentially degraded non-fibrillar collagens such as collagen IV, which has a small impact on passive stiffness [1, 10, 45]. Other considerations that could explain how collagenase would reduce passive stiffness when administered ex vivo, but not in vivo, are related to the in vivo physiological environment. During the first 24 h after injection, muscles exposed to CCH were consistently loaded whenever the mice were walking or standing. This constant tension on the collagen matrix within muscle makes it more difficult for collagenase to digest fibrillar collagens since collagen fibers under tension are harder to degrade [26, 46, 47]. Additionally, muscle is highly vascularized, and this allows soluble factors to be transferred and filtered out of the muscle. While we have evidence that CCH exerted an effect on the muscle, it is possible that filtration of CCH out of the muscle prevented sufficient degradation of fibrillar collagens to induce a drop in passive stiffness. Future studies should use purified collagenases that specifically target fibrillar collagens [19, 23, 24] to reduce muscle passive stiffness.

We expected that digesting the fibrotic collagen matrix would be neutral or beneficial to muscle strength at the end of the 7-day period possibly due to removal of restrictive fibrotic ECM and improved force transduction through the ECM. However, we observed that CCH did not improve ankle plantarflexion strength in D2.*mdx* mice and acutely reduced it in WT mice. This was paired with concomitant declines in EDL specific tension in both WT and D2.*mdx* mice following CCH. Interestingly, early drops in muscle strength after CCH were present before a change in muscle fiber size, demonstrating that declines in muscle strength were due to degradation of the ECM. Since fibrillar collagens are important to contractile force transmission [12, 48, 49], their degradation by CCH could reduce active mechanical properties. Nevertheless, collagen IV networks within the basal lamina are vital to muscle fiber stability and transduction of force through the ECM [2, 15, 50]. Within 24 h of CCH injections, collagen IV layer thickness was reduced, coinciding with drops in muscle strength. Interestingly, collagen IV layer thickness was increased 7 days following CCH injections, which phenocopies the muscle that lacks laminin, sarcoglycans, or other components essential to the sarcolemma-matrix interface [51–55]. Thus, it is evident that CCH impaired basal laminar stability and ECM force conducting properties, inducing early deficits in muscle strength. Similarly, the lack of increased susceptibility to eccentric contraction induced damage in CCH injected muscles likely reflects the instability of the sarcolemmal-matrix interface. The unstable muscle fiber-ECM connection likely damaged the muscles in vivo prior to the eccentric contraction protocols, which would explain their weakness and lack of force drop during the protocol. In another vein, if drops in muscle strength from CCH are derived from myofiber instability and poor ECM force transmission, the lack of change in ankle plantarflexion strength of D2.*mdx* mice following CCH could be explained by both myofiber instability and poor ECM force transmission at baseline [12, 38]. Therefore, it follows that CCH did not reduce contractile force transmission in the D2.*mdx* ankle plantarflexors to the same degree as the WT.

Another potential mechanism of CCH induced muscle weakness are altered tendon ECM integrity and mechanics, which could result in hindered force transmission between muscle and bone [19, 56]. The collagenous matrix within tendon is denser, more cross-linked, and stiffer than the matrix within muscle [7, 57, 58], which would make digestion of the tendon ECM more difficult. Even so, a decline in tendon stiffness could lead to a lower force readout in the ankle plantarflexion torque experiments. However, changes in tendon mechanics would not affect the specific tension measurements in the ex vivo muscle mechanics experiments, where muscles were tied at the myotendinous junctions. These declines in ex vivo muscle strength might be related to increased effect of CCH on smaller muscles compared to larger muscles, in which the dose or volume of CCH may not have been enough to exert a comparable effect. Greater ECM digestion by CCH likely induced greater myofiber instability at the interface of the matrix and sarcolemma within the EDL, reducing its ex vivo specific force. Thus, we postulate that early reductions in muscular strength following CCH are due to mechanical changes in the sarcolemma-matrix interface and the force-conducting properties of the muscle and tendon ECM.

During our experiments, we noticed that the limb that received CCH swelled and appeared red within 24 h of injection. Also, upon dissection muscles from the limb receiving CCH had a consistent red coloration that did not disappear when washed with Ringer’s solution. Thus, we suspected that CCH induced inflammation and intramuscular bleeding due to its bacterial epitopes and degradation of collagen IV networks, respectively. To our surprise, when we investigated immune infiltration in the TA, we did not observe an increase in CD45 + cells in CCH injected muscles. This may have been due to the inflammation subsiding prior to dissection or immune infiltration around, but not within, the muscle itself. When we followed this up with immunohistochemistry for intramuscular bleeding in the TA, we observed that CCH increased the fibrinogen content within muscle but was not associated with altered capillary abundance. Thus, we infer that CCH digested the collagen IV within blood vessel walls to induce intramuscular bleeding but did not induce further vascularization of the muscle [40, 59].

While CCH was not successful in reducing muscle fibrosis in this study, our results inform future strategies to combat muscular fibrosis by targeting the ECM using collagenases or other matrix-digesting compounds. We outline three strategies to mitigate the possibility of inducing a pathological effect when using collagenase to study fibrosis. First, ensure that the selected compound is specific to the target of interest. Prior to our study, we considered that our selected collagenase would degrade non-fibrillar collagens to some degree, but we did not expect this to be the major effect of CCH. FDA-approved compounds containing CCH have demonstrated specificity for fibrillar collagens [24, 60]. Clearly, specificity of the compound has an impact and off-target effects to the ECM result in deficits in muscle strength. Additionally, while CCH is used to target collagen-rich cords in Dupuytren’s contracture and Peyronie’s disease, we used CCH in muscle to target increased intramuscular collagen within the perimysial space, a region of the muscle where structural collagen along with blood vessels and neural elements reside. The accessibility of this collagenous region and its close proximity to collagenase-sensitive elements make specificity even more important for the use of CCH in muscle. Second, consider how targeting ECM in different disease models may impact the expected outcomes. In models of dystrophy the pathology stems from intrinsic deficits in muscle fiber function while matrix functional deficits are secondary [14, 61, 62]. Thus, even if CCH reduced fibrosis in dystrophic muscle, the muscle fibers could still incur significant contraction-induced damage leading to chronic remodeling and a return to the fibrotic state. Selecting other models of muscle fibrosis or injury that retain a capacity for muscle regeneration less susceptible to damage might respond better to exogenous CCH or other compounds that target ECM components. Finally, the use of combinatorial approaches that feature pharmaceutical and physical therapeutic intervention might be more effective than either one alone. In the case of Peyronie’s disease and Dupuytren’s contracture, for which CCH injections are approved, physical manipulation at the site of injury is typically performed within days after the injection [20, 60]. Indubitably, skeletal muscle is responsive to changes in activity, which alter the outcomes of ECM and muscle fiber remodeling in the context of injury and disease [63–65]. Again, considering the model of interest is important to determining the choice of physical therapy, since the response to muscle contractions following a large digestion of the ECM might be detrimental to dystrophic muscles but helpful to wildtype controls.

## Conclusions

Overall, we show that intramuscular injections of non-specific CCH have harsh effects on muscle strength and integrity. However, purified forms of CCH that only target fibrillar collagens could be effective at reducing fibrosis and stiffness in skeletal muscle. Additional study is needed to ascertain the correct dosage, paired physical intervention, and time course that would provide a therapeutic benefit to people with muscle fibrosis.

## Supplementary Information


Supplementary Material 1.


## Data Availability

All data are available in the main text or the supplementary materials with individual points. Data tables are available from the corresponding author upon reasonable request.

## Abbreviations

ECM: Extracellular matrix
DMD: Duchenne muscular dystrophy
CCH: Collagenase Clostridium histolyticum
CP: Cerebral palsy
HBSS: Hank’s Balanced Salt Solution
TA: Tibialis anterior
EDL: Extensor digitorum longus
GA: Gastrocnemius
QU: Quadriceps
WT: Wildtype
EBD: Evan’s blue dye
OCT: Optimal cutting temperature
L_o_: Optimum length
PCSA: Physiological cross-sectional area
L_m_: Muscle length
PFA: Paraformaldehyde
PBS: Phosphate buffered saline
BSA: Bovine serum albumin
ANOVA: Analysis of variance
