# Supplementary material for: Targeting muscle fibrosis using non-specific collagenase injections destabilizes the basal lamina and induces matrix remodeling
Source: Skelet Muscle. 2026 Jun 19;16:33. doi: 10.1186/s13395-026-00432-7 (PMC13536639; doi:10.1186/s13395-026-00432-7)
Supplement: Supplementary file 1 [file 13395_2026_432_MOESM1_ESM.pdf]

Supplementary Figures

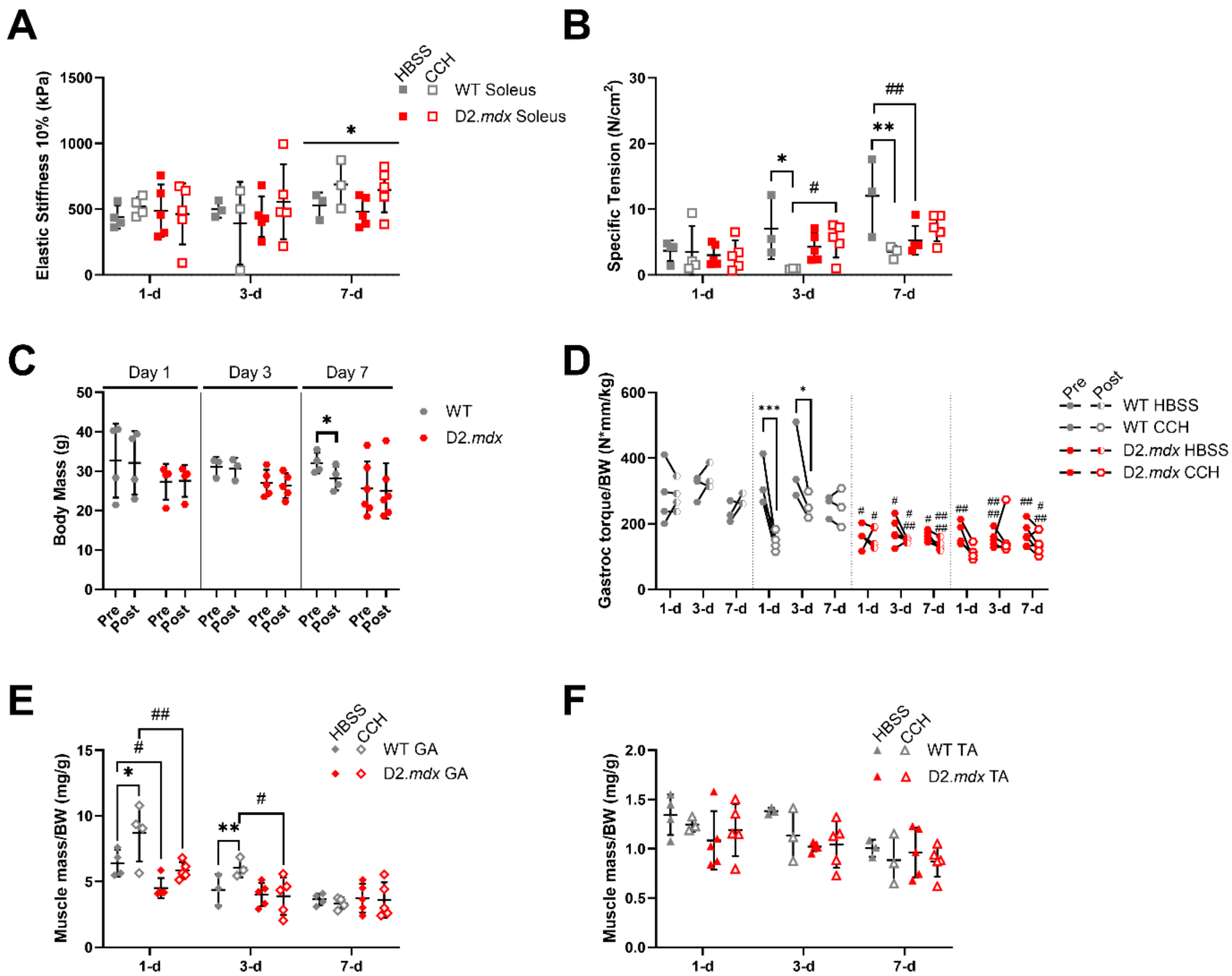

Supplemental Figure 1. Passive and active muscle mechanical properties.
(A) CCH injected solei were stiffer at 7-days post-injection than saline injected. **(B)** Specific tension was higher in the WT soleus compared to D2.*mdx* at 3- and 7-days post-injection. CCH injected WT solei were weaker than saline injected. **(C)** There were no large (>20%) changes in body mass across the time course. **(D)** Plantarflexion torque was reduced in WT muscles by CCH injection. D2.*mdx* plantarflexors were weaker than WT across the time course. **(E)** WT GA muscles had greater normalized muscle mass following CCH injections. D2.*mdx* GA muscles tended to be smaller than WT. **(F)** TA muscle mass did not exhibit changes in normalized muscle mass with CCH injections. Significance by CCH: *p<0.05, **p<0.01, ***p<0.001; significance by genotype: $^{\#}$p<0.05, $^{\#\#}$p<0.01.

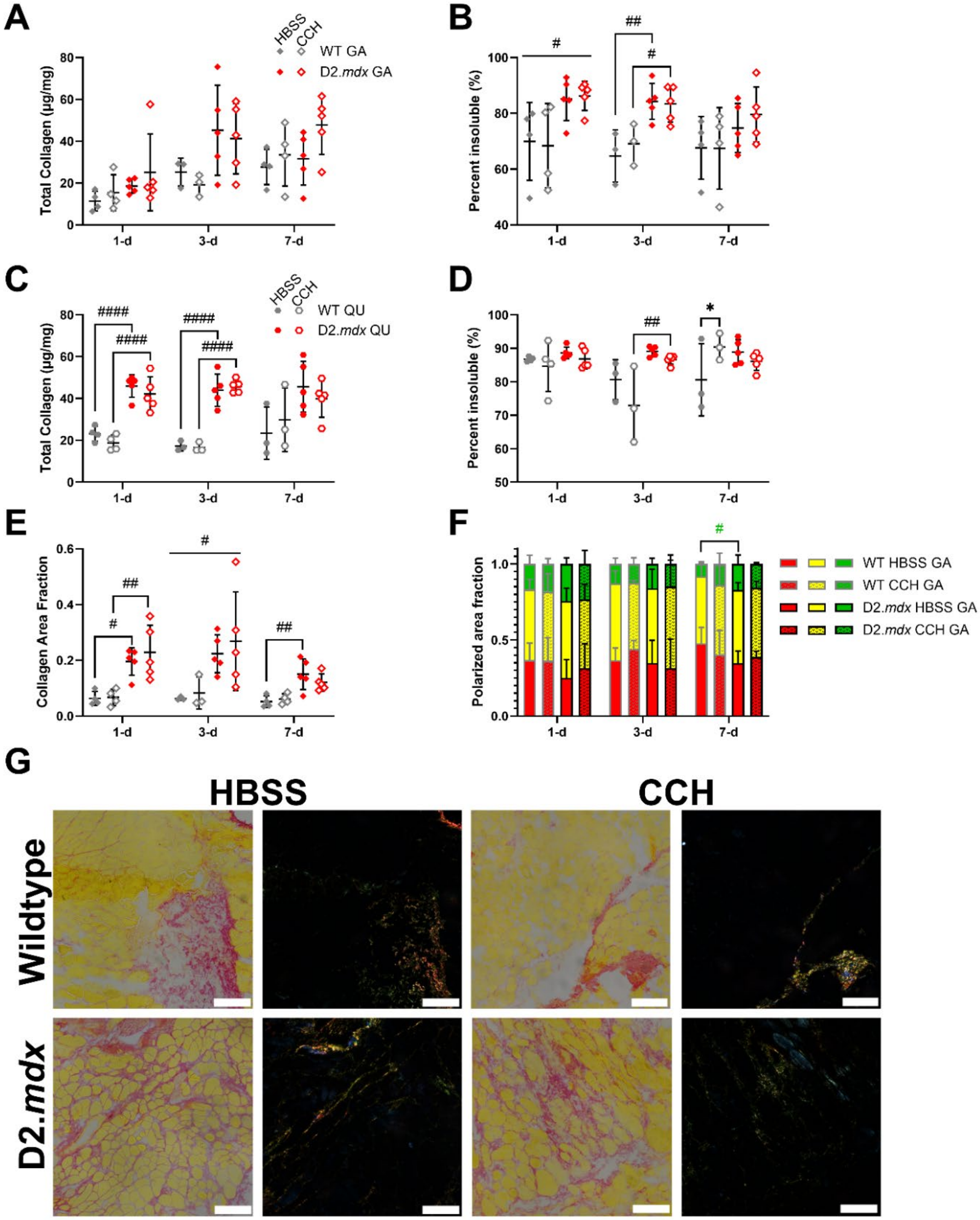

Supplemental Figure 2. Collagen characteristics in gastrocnemius and quadriceps muscles.
(A) Total collagen content was not significantly different across gastrocnemius (GA) muscles. **(B)** D2.*mdx* GA muscles tended to have more cross-linked collagen than WT. **(C)** Total collagen content was higher in D2.*mdx* quadriceps (QU) muscles compared to WT. There were no effects of CCH on QU collagen content. **(D)** D2.*mdx* QU muscles had more cross-linked collagen than WT at the day-3 timepoint. **(E)** D2.*mdx* GA muscles had higher collagen area fractions than WT. **(F)** D2.*mdx* GA muscles had more loosely packed collagen than WT 7-days after CCH. **(G)** Representative images of Sirius Red stained cross-sections of GA muscles. Scale bar=200µm. Flat bars represent significant main effects from two-way ANOVAs or mixed effects analyses. Bracketed bars represent significance from Sidak's multiple comparisons post-hoc tests. Significance by CCH: *p<0.05; significance by genotype: $^{\#}p<0.05$, $^{\#\#}p<0.01$, $^{\#\#\#\#}p<0.0001$.

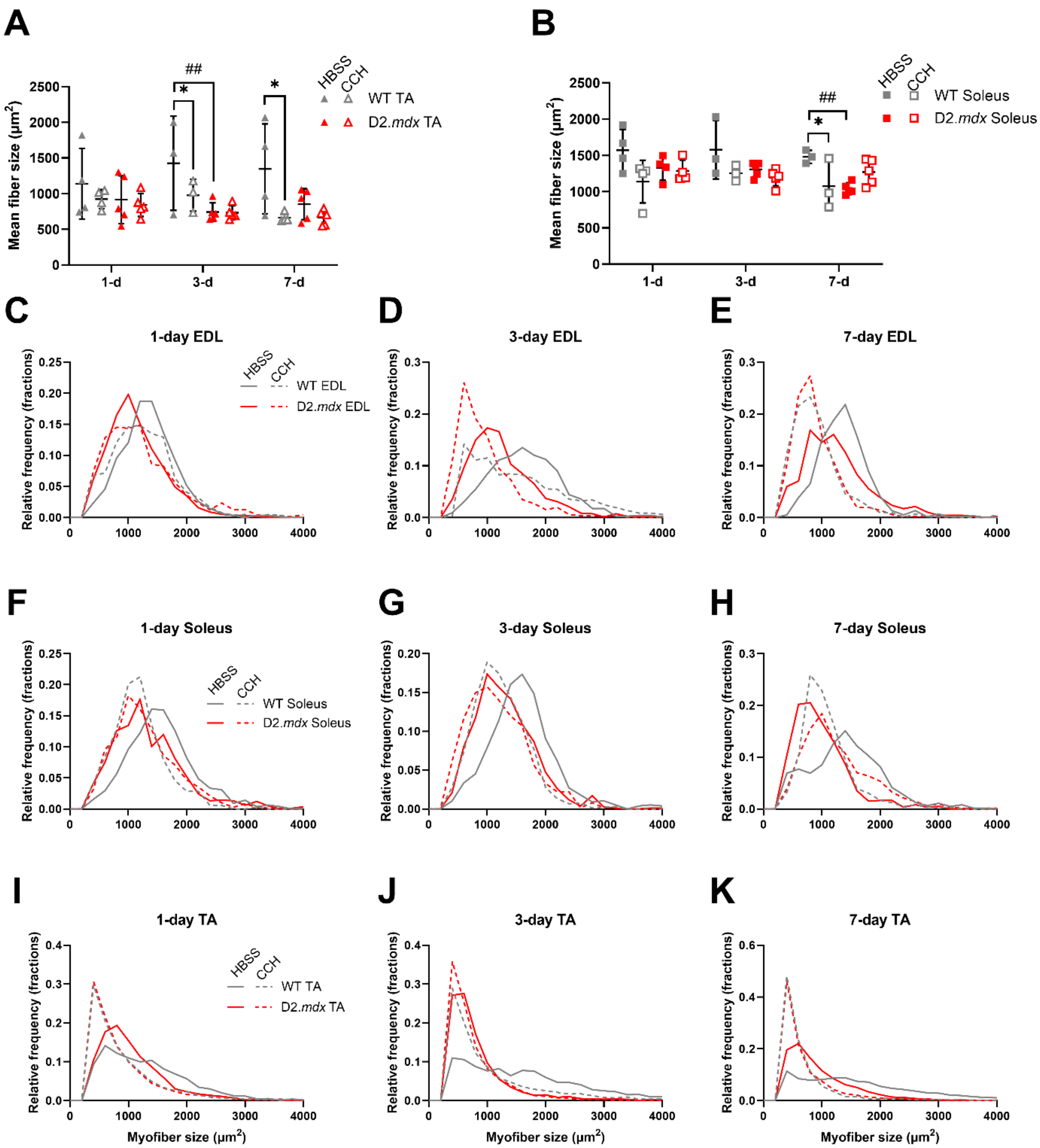

Supplemental Figure 3. Muscle fiber size.

(A) CCH reduced muscle fiber size in the WT TA after 3 days. **(B)** CCH reduced the muscle fiber size of the WT soleus after 7 days. **(C-E)** Histograms showing fiber size distributions in the EDL across the time course. **(F-H)** Histograms showing fiber size distributions in the soleus across the time course. **(I-K)** Histograms showing fiber size distributions in the TA across the time course. Flat bars represent significant main effects from two-way ANOVAs or mixed effect analyses. Bracketed bars represent significance from Sidak's multiple comparisons post-hoc tests. Significance by CCH: *p<0.05, **p<0.01, ***p<0.001; significance by genotype: $^{\#}$p<0.05, $^{\#\#}$p<0.01, $^{\#\#\#}$p<0.001, $^{\#\#\#\#}$p<0.0001.

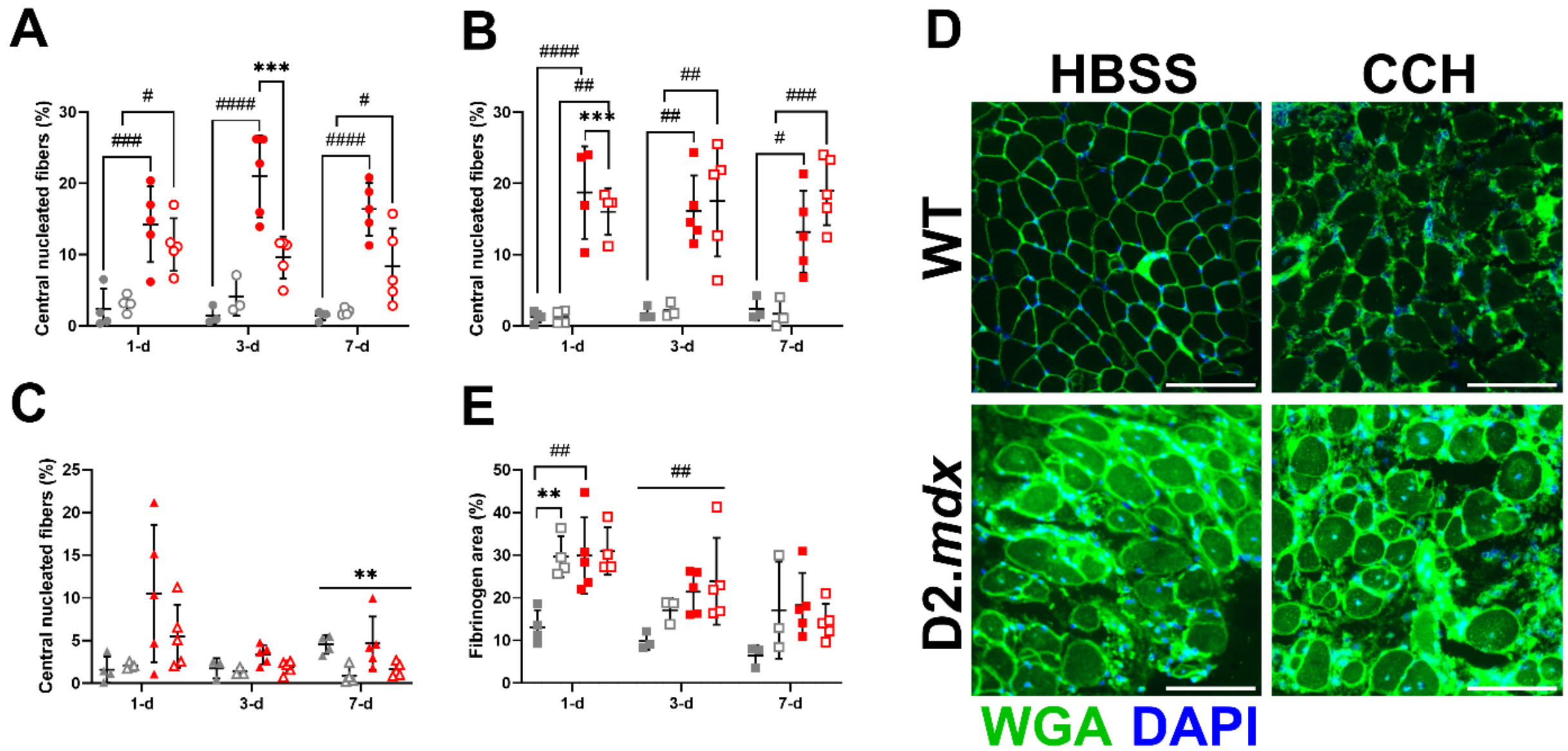

Supplemental Figure 4. Central nucleation and fibrinogen area.

(A-B) Central nucleation was more prevalent in D2.*mdx* EDL and soleus muscles compared to WT. **(C)** CCH injected TA muscles had reduced central nucleation after 7 days. **(D)** Representative images of centrally nucleated fibers in TA muscles. Wheat germ agglutinin (WGA) shown in green, nuclei (DAPI) shown in blue. Scale bar=150µm. **(E)** D2.*mdx* soleus muscles had higher fibrinogen area compared to WT. CCH increased fibrinogen area in the soleus 24 hours after injection. Flat bars represent significant main effects from two-way ANOVAs or mixed effect analyses. Bracketed bars represent significance from Sidak's multiple comparisons post-hoc tests. Significance by CCH: *p<0.05, **p<0.01, ***p<0.001; significance by genotype: $^{\#}p<0.05$, $^{\#\#}p<0.01$, $^{\#\#\#}p<0.001$, $^{\#\#\#\#}p<0.0001$.

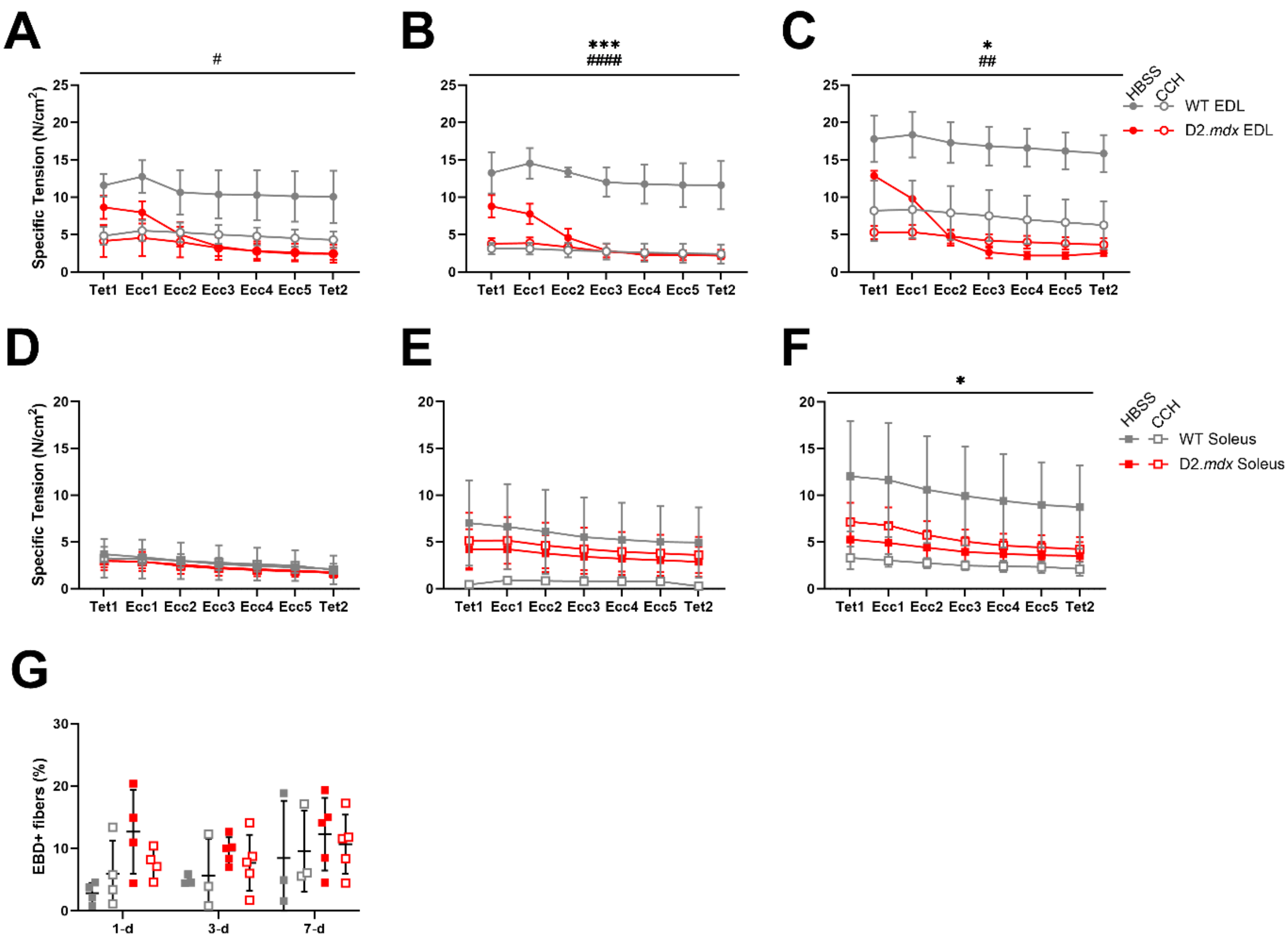

Supplemental Figure 5. Eccentric contraction induced damage in EDL and soleus muscles.
(A-C) Specific tension of D2.*mdx* muscles decline with eccentric contractions. CCH injected EDL muscles were weaker than saline injected. **(D-F)** Soleus muscles exhibited less eccentric contraction induced damage related to dystrophy. **(G)** Percentage of Evans blue dye (EBD) positive fibers was not significantly changed across groups in the soleus. Flat bars represent significant main effects from two-way ANOVAs or mixed effect analyses. Bracketed bars represent significance from Sidak's multiple comparisons post-hoc tests. Significance by CCH: *$p<0.05$, ***$p<0.001$; significance by genotype: $^{\#}p<0.05$, $^{\#\#}p<0.01$, $^{\#\#\#\#}p<0.0001$.
